# Supplementary material for: Frequency-Dependent Sternheimer Linear-Response Formalism for Strongly Coupled Light–Matter Systems
Source: J Chem Theory Comput. 2022 Jun 8;18(7):4354–65. doi: 10.1021/acs.jctc.2c00076 (PMC9281401; doi:10.1021/acs.jctc.2c00076)
Supplement: Supplementary file 1 — ct2c00076_si_001.pdf [file ct2c00076_si_001.pdf]

Frequency-Dependent Sternheimer Linear-Response Formalism for 2 Strongly Coupled Light–Matter Systems

Davis M. Welakuh,[*,†,‡] Johannes Flick,[*,¶] Michael Ruggenthaler,[*,†] Heiko Appel,[*,†] and Angel Rubio[*,†,¶]

†*Max Planck Institute for the Structure and Dynamics of Matter and Center for Free-Electron Laser Science & Department of Physics, Luruper Chaussee 149, 22761 Hamburg, Germany*

‡*Harvard John A. Paulson School Of Engineering And Applied Sciences, Harvard University, Cambridge, Massachusetts 02138, USA*

¶*Center for Computational Quantum Physics, Flatiron Institute, 162 5th Avenue, New York, NY 10010, USA*

E-mail: dwelakuh@seas.harvard.edu; jflick@flatironinstitute.org; michael.ruggenthaler@mpsd.mpg.de; heiko.appel@mpsd.mpg.de; angel.rubio@mpsd.mpg.de

A Photo-absorption cross-section of the azulene molecule

To describe the azulene molecule studied in Sec. 4, we compute the electronic structure of the azulene molecule in real space using the same setup as that in Ref.[1] for the benzene molecule. That is, the simulation box is a cylinder of length 8 Å with a radius of 6 Å in the x-y plane and a spacing $\Delta x = 0.22$ Å . We treat the core electrons of the carbon and hydrogen atoms using the local-density approximation (LDA) Troullier-Martins pseudopotentials.[2] Since the

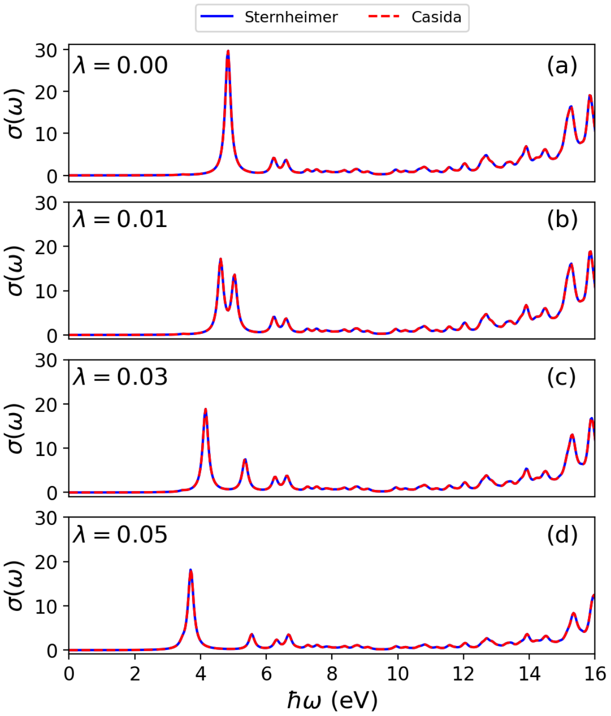

Figure 1: Comparison of the photo-absorption cross-section of an azulene molecule using the electron-photon Sternheimer and Casida methods. (a) Absorption cross-section in free space (i.e. $\lambda_\alpha = 0$). Coupling the cavity mode resonantly to the $\pi - \pi^*$ transition and increasing the coupling strength continuously as in (b) to (d) leads to a Rabi splitting into lower and upper polariton branches. Both approaches are in good agreement for the computed spectra.

Sternheimer method considers only occupied orbitals, the 48 valence electrons that amounts to 24 doubly occupied orbitals are used in the computation. When we use the Casida approach, we include 500 unoccupied states which amounts to $N_v * N_c = 24 * 500 = 12000$ pairs of occupied-unoccupied states. Also, we compute the electronic structure for the LiH molecule using a simulation box which is a cylinder of length 20 Å with a radius of 6 Å in the x-y plane and a spacing $\Delta x = 0.14$ Å . The core electrons of lithium and hydrogen atoms are also treated using the LDA Troullier-Martins pseudopotentials.[2] To account for the electron-electron and electron-photon interaction in the linear-response QEDFT framework,[1] we apply the adiabatic LDA (ALDA) to the Hartree exchange-correlation kernel (i.e. $f^n_{\mathrm{Hxc}} \rightarrow f^n_{\mathrm{Hxc,ALDA}}$) and the photon random-phase approximation to the photon exchange-correlation kernels (i.e. $f^n_{\mathrm{pxc}}, f^{q_\alpha}_{\mathrm{pxc}} \rightarrow f^n_{\mathrm{p}}, f^{q_\alpha}_{\mathrm{p}}$), respectively. We compute the photo-absorption cross-

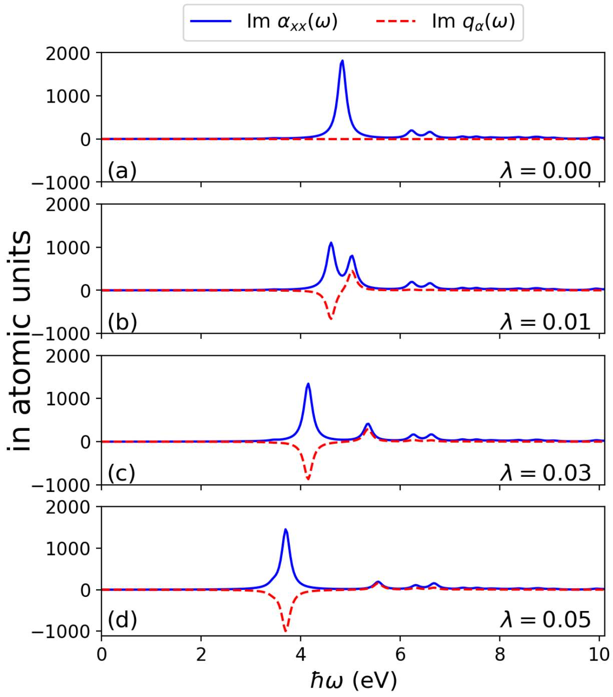

Figure 2: The spectrum of the imaginary parts of the polarizability and photon displacement coordinate of an azulene molecule in free space and in an optical cavity. (a) The free space case where light decouples from matter and there is no response from the photon subsystem. Panels (b)-(d) shows the light-matter coupled case where both observables are shown to have the same excitation energies but different oscillator strengths.

section of the azulene molecule in free space and inside the high-Q optical cavity and the results are shown in Fig. (4). This result is related to the imaginary part of Fig. (2) and it shows more excitations at higher energies.

In the linear-response setting of non-relativistic QED in the length gauge,[1] the response quantities that arise have the same excitation energies but different oscillator strengths as shown in Fig. (8) for two observables of interest. This is because their respective response functions have the same excitation energies and different oscillator strengths.[1]

References

(1) Flick, J.; Welakuh, D. M.; Ruggenthaler, M.; Appel, H.; Rubio, A. Light-Matter Response in Nonrelativistic Quantum Electrodynamics. *ACS Photonics* **2019**, *6*, 2757–2778.

(2) Troullier, N.; Martins, J. L. Efficient pseudopotentials for plane-wave calculations. *Phys. Rev. B* **1991**, *43*, 1993–2006.
